# The Emergence of (Male) Eating Disorders as a Clinical Entity

**DOI:** 10.1111/1467-9566.70045

**Published:** 2025-05-13

**Authors:** Piotr Maron

**Affiliations:** ^1^ Faculty of Humanities, Arts and Social Sciences University of Exeter Exter UK

**Keywords:** care, gender, male eating disorders, maleness

## Abstract

Psychiatric and psychological research has confirmed that less than 1% of the research on eating disorders is focused on males. However, for the first time, the occurrence of eating disorders is reportedly growing faster among the male population. Nevertheless, men still are more likely to stay undiagnosed. This paper bridges this gap and offers an analysis of male eating disorders (MEDs) and particularly drawing from a feminist technoscience perspective, it examines how male eating disorders are made up in clinical practices and encounters. Specifically, in this paper, I investigate the different ways by which male eating disorders emerge as a situated matter of concern and object of clinical care. In other words, I explore the ‘making present’ of the male and maleness in the clinical practices treating eating disorders in the Australian healthcare system. Based on the data from 25 semi‐structured, qualitative interviews with clinicians, the paper draws out how care in relation to eating disorders is organised and, specifically, how the enactment of a female/male binary mobilised in clinicians' accounts of clinical practices may act to constrain care. Finally, I demonstrate how care practices could attend to male eating disorders differently in a more sensitive and intersectional way.

## 1 Introduction

Male eating disorders (MEDs) are becoming an important public health concern in Australia and globally. However, despite increased media attention (Boltje 2017; Bain 2012; Callari 2022) and policy investment in Australia (Department of Health and Aged Care 2019), MEDs remain under‐researched. Indeed, psychiatric and psychological research has confirmed that less than 1% of the research on eating disorders (EDs) is focused on males. Hence, ‘maleness’ is construed as a rare occurrence in the eating disorders literature (Murray et al. 2014; Murray and Touyz 2013; Thapliyal et al. 2020). However, approximately one‐third of individuals diagnosed with an eating disorder are now boys and men (Mitchison et al. 2020); nevertheless, men are still more likely to stay undiagnosed (Lavender et al. 2017).

In terms of diagnostic criteria, the Diagnostic and Statistical Manual of Mental Disorders (DSM‐V) addressed male experiences of eating disorders. Specifically, amenorrhoea was removed as a necessary diagnostic criterion for anorexia in the DSM. Furthermore, the criteria for bulimia nervosa (BN) were adjusted to better align with the experiences of men, who tend to report fewer instances of objective binge eating episodes. Additionally, new diagnostic categories were introduced to encompass a broader range of male experiences with eating disorders. For example, binge eating disorder was recognised as a distinct condition in the DSM‐V. This diagnostic criterion appears to more accurately reflect male experiences, as men represent a larger proportion of binge eating disorder cases compared to those of anorexia nervosa or bulimia nervosa (Spratt et al. 2022; Schaumberg et al. 2017). Moreover, binge eating disorder is regarded as the most prevalent among all eating disorders (Santomauro et al. 2021). In relation to the DSM classification, males may engage in behaviours such as purging or binging but interpret them as coping mechanisms rather than recognising them as symptoms of a specific disorder (Räisänen and Hunt 2014), which can complicate the diagnostic process.

Within qualitative social studies of health, a systematic review on MEDs identified only four papers that investigated this issue (Thapliyal and Hay 2014). A subsequent review in 2021 pointed to only nine such studies (Richardson and Paslakis 2021). The UK‐based charity BEAT highlights that although studies exist on the prevalence of eating disorders among men using DSM‐IV criteria, there is a notable lack of evidence regarding the prevalence of MEDs based on DSM‐V criteria.

Noting that MEDs are insufficiently understood in qualitative social studies of health (Arnow et al. 2017; LaMarre et al. 2022a, 2022b), in this paper, I bridge this research gap, and drawing from a feminist technoscience perspective, I investigate the different ways by which male eating disorders emerge as a situated object of clinical care. In other words, I explore the ‘making present’ of the male and maleness in the clinical practices treating EDs in the Australian healthcare system.

My analysis falls into two parts. First, I explore various objects and sites before this phenomenon emerges as recognised or diagnosed as a ‘male eating disorder’ in clinical practice. Second, I examine how the disorder imagined by practitioners to exist ‘out there’—in part, imagined as ‘female’—resists being ‘brought into’ the clinical space. In doing so, I draw out how care in relation to eating disorders is organised and, specifically, how the enactment of a female/male binary mobilised in practitioners' accounts of clinical practices may act to constrain care. Finally, inspired by the approach to care proposed by STS scholars (Martin et al. 2015; Puig de la Bellacasa 2011), I demonstrate how care practices could attend to male eating disorders differently in more sensitive and intersectional ways, engaging with the question: How to care differently?

## 2 Approach

To capture how things are and emerge in the world or how MEDs are and emerge inside and outside clinical settings, I follow Barad's (2007) definition of relationality. That is, the fundamental way things are in the world derives from relationships maintained with other things. In other words, the existence of objects is never predefined by external characteristics (Harrison and Olofsson 2016). Objects and phenomena do not exist independently, waiting to be unearthed or discovered—they emerge. Furthermore, knowing about objects and reality is accomplished through the intra‐actions that enable various materialisations in the world. That is how matter and the world come to matter (Barad 2007). Hence, knowledge about things produces effects here and now, for instance, during clinical encounters. That is how our reality becomes and how it matters.

This paper discusses how MEDs become recognised and attended to as a clinical entity in care settings. Drawing on recent research on care in STS and feminist technoscience (Jerak‐Zuiderent 2015; Latimer 2018; Maron 2022; Mol 2008; Rhodes et al. 2018; Singleton and Mee 2017) and in line with the recent questions asked within the STS community about what care is, what the future of care is, who cares or how to care (Lindén and Lydahl 2021), I adhere to a relational conception of care, as proposed by Puig de la Bellacasa (2011, 2017). In relation to MEDs, I am interested in how care and its materialities (Buse et al. 2018; Latimer 2018) emerge in clinical settings. Thinking with care entails that we think with ‘ontological heterogeneity’ and multiplicity. Subsequently, it allows me to see care through care practices in sociomaterial spaces, such as treatment centres, as care also ‘touches’ and ‘moves’ everyone and everything around (Mol et al. 2010).

The analysis in this paper is based upon 25 (*n* = 25) semi‐structured, in‐depth, qualitative interviews with clinicians involved in the treatment of eating disorders within the Australian public health sector and the equivalent of the national health service—Medicare. That is why I interviewed clinical psychologists (9), general practitioners (2), mental health nurses (3), dieticians (8), an exercise physiologist (1) and psychiatrists (2). Recruiting clinicians from different clinical fields enriched the depth of my data and provided a more robust perspective on the topic. The interviews were conducted between July and December 2021. Because of COVID‐19 restrictions, they were conducted over Zoom in Australia. The Zoom format enabled me to recruit participants from across all Australian states, offering a broader and more comprehensive perspective on clinical practices surrounding male eating disorders. I was also mindful that this format could be more restrictive to distil clinical practices in relation to MEDs. An interview schedule was designed to navigate and inform a smooth interviewing process with the participants. After reviewing the literature on MEDs, I anticipated that clinicians would have a very limited number of male patients—a prediction that was confirmed by my interviewees. Therefore, my key sampling aim was to interview health professionals involved in treating eating disorders in Australia, having at least one male patient in accordance with the eating disorder treatment and management plan introduced in 2019 (Maguire et al. 2022); thus, my research sample spans six medical professions. Considering the theoretical framework informing my research and the qualitative character of the study, it was important that the approach to analysis emphasised a commitment to relational ontology (Barad 2007; Law 2004). This is why, to analyse the interviews, I used a data analysis process informed by constructivist grounded theory approach principles (Charmaz 2006). The ontoepistemological premise of Charmaz's version of grounded theory linked with the feminist technoscience theoretical commitments underpinning this research informed how I approached my interview data. Such an approach allowed me to go beyond the mere spoken word to ‘map human and nonhuman processes as always in becoming’ (Dennis 2019) and form a research assemblage (Fox and Alldred 2015a, 2015b).

To answer this paper's questions, I also draw on the specific conceptualisation of the notion of practice within the field of STS (Law and Singleton 2005; Mol et al. 2010). That is, practices are not located outside reality; they produce it. Saying that realities are created in practices entails seeing the relationships and entanglements that practices initiate. I show how MEDs become through practices in relation to an assemblage of bodies, places and clinical work. In this paper, I am particularly attuned to how clinical practices underscore the emergence of MEDs in clinical settings because the relation between medical knowledge, bodies and people is based upon practices.

Furthermore, through interview data, I aimed to follow clinical practices ‘around’, examining how they are generative in creating objects of interest—specifically, how, for instance, objects were brought to life by situating the encounter in the participants' workplace or how, through interviews, I noticed emerging materialities. Moreover, I wanted to capture how clinicians attend to patients in the moments of their encounters. However, to access practices in greater detail and to have a better sense of the clinical spaces themselves, I applied an ethnographically inspired approach to conduct my interviews. For instance, I asked the participants to show me assessment tools such as weight scales and charts or medical textbooks. I also asked my interviewees to show me the medical guidelines they use to assess and diagnose their patients. Hence, the interviews exceeded mere verbal talk, mobilising objects and texts in the moment. This way, I could immerse myself in the moment during our discussion and later—reflecting while analysing—I could re‐experience these encounters to gain a more distinctive analytical perspective.

## 3 Analysis

### 3.1 Male Eating Disorders ‘Outside’ the Clinical Setting

Coming into interviewing healthcare professionals about male eating disorders, I was curious how much experience they would have with male cases. While talking to clinicians, it was clear that MEDs are only a small proportion of the total number of cases they see. For instance, John, a dietician working on eating disorders for the last 5 years, said that he had seen only two male patients. Katrina, another dietician working on eating disorders, told me that approximately 5% of her patients are male. Many of those participating in interviews accentuated or qualified their partial expertise in relation to MEDs: ‘I cannot generalise’; ‘It is anecdotal’; ‘I don't have evidence‐based research to support what I have observed’. This implies that clinicians know the disorder's existence, but its visibility is obscured. Lastly, the enactment of the disorder existing ‘outside’ a clinical setting materialised in responses to my interview email invitations. Although greeted with interest, some refused to participate, highlighting the scarcity of patients or emphasising their very limited experience with male eating disorders. The limited direct engagement with MEDs in some cases shifted accounts into the realm of *imaginaries* in relation to male patients with an eating disorder and how this disorder might present outside the clinical setting. In relation to the ‘outside’ and ‘inside’, clinicians often emphasised the particular tension between what they ‘think’ happens outside and what they see in their practices. One of the general practitioners, Jackie, a GP working on eating disorders for over 20 years, said:

I would say this even though we know that eating disorders in males is quite common as well, they don't present as frequently and they are far less likely to actually come in saying, “I think I have a problem. I think I might have an eating disorder”.

(Jackie)

Jackie construes male eating disorders as existing ‘out there’. She imagines MEDs as ‘quite common’ but infrequently made apparent to clinicians, in part because patients do not problematise themselves as in need of care. Jackie further accentuated the uncertainty of problematising or knowing an eating disorder as she imagines patients experiencing this: ‘I *think* I have a problem. I *think* I *might* have an eating disorder’ [emphasis added]. With this, she indicated that men may not realise that they may be at risk and may not know how to seek help. She imagined, therefore, that many men do not show up to see a specialist even if they are experiencing what might be later identified as an eating disorder. In other words, male eating disorders are imagined to be existing, ‘out there’, unrecognised until they are brought into the clinical space and *made more recognisable*.

In Jackie's story, a male eating disorder appears as a presumed entity existing outside the clinical realm by imagining what a male client could have said. Here, Kathrine, a clinical psychology registrar, told a story about one of her male patients in the clinical setting. Here, the matter of concern is presented in the clinic but is made more recognisable with difficulty:

I had a 60 year veteran, war veteran, I wasn’t seeing for an eating disorder and he clearly had a binge eating issue. But it wasn’t an issue for him. His weight and his stomach was an issue to him but it was joked about and laughed off. It wasn’t something he asked for help with.

(Kathrine)

Even though Kathrine noticed a problem, her patient had a rather light‐hearted approach to his body shape concerns. Kathrine, however, hinted that it was much more serious. Nevertheless, the patient was seeking clinical help for other reasons. Although the clinician saw binge eating symptoms as a matter of concern, the patient joked about his weight and his stomach, dismissing this as a clinical concern. Thus, when Kathrine said that he ‘clearly had a binge eating issue’, her account enacts the disorder as something *recognised* but not actionable for her:

Yes, he just saw it as something that he lived with and something he did and something to make jokes about. Not something to ask for help with. And if I offered it, he was like, oh no no.

(Kathrine)

For Kathrine, this situation is difficult because, as a clinician, she could not intervene in something that her patient does not see as a clinical problem.

Alan, an early‐career researcher and clinical psychologist working in a public hospital, described another situation where the matter of concern ‘out there’ might *become recognisable* in a clinical sense, yet a patient did not acknowledge a problem:

I am thinking about one male that I worked with in private practice where the concern for his health and wellbeing was coming from his family and not from him, which is already a really challenging thing because you know when you are working with someone you are working with their motivation, their goals. When the reason that they are seeing you is because their father, mother, and sister are really worried about them because they have all these challenges with their eating and they are throwing up and spending lots of time at the gym, what you are dealing with is that they just want to be continuing to do that.

(Alan)

However, in Alan's story, the problem is *recognised* differently than in Kathrine's case: as a matter of concern made in relation among the client, their family and the clinician. Here, the patient, again, did not see these practices as a problem in need of clinical intervention, but the family circle noticed what they regarded as concerning signs. Ultimately, Alan, like Kathrine, found it difficult to intervene when working with a patient who did not acknowledge that they might be at risk in the ways that clinical ‘disordering’ requires. Not seeing a problem or not coming to see a clinician speaks to how what later may be enacted as ‘male eating disorders’ may or may not exist before the concern is translated into a clinical discourse and made recognisable through those clinical sites and practices. Outside the medical setting, what may come to be constituted as MEDs is fluid; until this constellation of concerns or symptoms is translated into a medical system and enacted as a disorder through diagnosis, they exist as ‘something else’ (Callon 1984; Mol 2002). By that I also do not mean that they could be literally anything. The matter of concern remains real even if not recognised as a clinical object by a veteran or Alan's client. It is just not yet made as an actionable clinical object—an object of management and clinical care.

Clinicians admitted that the problems the patient might have (Jackie) or had (Kathrine and Alan) were not able to be considered or treated as ‘eating disorders’. Therefore, clinicians need to navigate and negotiate how to attend to or intervene in these complex moments, that is, to help make a matter of concern into an object of clinical intervention and care. Hence, the problem pertains not only to knowing but to making recognisable and actionable the matter of concern through clinical practices. The issue concerns what happens in ‘actually coming to treatment’ as Annie mentioned.

How eating disorders might be made recognisable and as objects of clinical care was thought to differ for men and women. In her interview, Annie, a psychiatrist working with eating disorders for more than 30 years, highlighted how the process of making a ‘disorder’ more recognisable is shaped by particular (gendered) situations:

Occasionally it might be a physician that they might have gone to for some complication, but for girls quite often it’s picked up in schools by general practitioners and often by parents, but I think for boys it seems to take longer. For girls, you know somebody who is of normal weight suddenly appears to be like skin and bone, even then it’s taken a long time, but I think for boys, I think often there is a lot of questioning around it and doubt about it before they actually come to treatment.

(Annie)

Collete, a clinical psychologist and eating disorders researcher, also speculated about unrecognised (undiagnosed) disorders for men: *‘I think* *that probably there are lot more males with anorexia and restrictive eating disorders than probably we would be aware of’.* (Colette) Additionally, Rebeka had a similar observation: *‘So, I am sure there is more going under the radar than we realize’.* (Rebeka) Hence, whatever is affecting men ‘outside’ is imagined as present, but it is not yet ‘an eating disorder’—it is still disconnected from the medical system. The small number of male cases that Colette sees in her practice does not mean there are not more, but they are not yet made recognisable and treatable, whereas they remain outside her clinical scope. She speculates that the occurrence of MEDs is much more prevalent, enacting it as something that is, in fact, happening on a larger scale in the community. However, in the community, these matters of concern exist as something other than ‘men at risk of having an eating disorder’. Terese, a clinical psychologist, spoke in more detail about this, expressing concern about low case numbers of male clients:

So, it's a real issue and the fact that I haven't had a referral for males with an eating disorder, knowing that binge eating disorders in males and females, is *fairly equivalent, or there about, so it's a real issue*.

(Terese)

By imagining equivalence in male and female cases, Terese's account enacts a concern that elides being made recognisable in the clinical space.

In this constellation of enactments, the object of MEDs is made more recognisable to a certain degree because it is imagined that it may eventually become a part of a healthcare system. In other words, MEDs existing ‘outside’ and ‘inside’ the clinical realm are connected to each other (Law and Singleton 2005) even though, seemingly, they exist separately.

So far, Alan, Colette, Kathrine and Rebeka painted a picture where clinicians are put in a peculiar situation in relation to the MEDs they imagine ‘out there’, whereby the issue may be partially recognised but difficult to act upon until it *becomes* recognisable through diagnosis in clinical practices. Now, I turn my analysis to consider how health professionals imagine barriers that impact MEDs becoming a clinical entity.

### 3.2 Emergence Through Barriers

In the accounts of Colette and Annie, as we saw above, men are imagined as experiencing healthcare services differently from women. Through their speculation, Colette and Annie both made connections to gender. Subsequently, different experiences in relation to gender make up the matter of concern differently for men and women. It is a situated concern. Although gender‐related healthcare access and how gender relations delimit care delivery for men and women are well explored in social studies of health (Gough 2007; Gough and Conner 2006; K. Hay et al. 2019), here I concentrate on how maleness, in itself, is enacted in participants' accounts as problematic in the clinical domain of eating disorders, materialising as a barrier for a successful care delivery. I do not conceptualise maleness within normative gender binaries. Instead, drawing on Barad’s framework, I approach maleness and masculinity as dynamic and emergent, examining how their becoming operates as a barrier within situated clinical encounters. For instance, Colette imagined men's experiences with the health system as follows:

I am not sure, maybe males don't necessarily get that kind of experience growing up and so it just feels like “I only go to the GP if I need a referral or if I am really sick and I need antibiotics.”

(Colette)

In Colette's account, it is presumed that men are less likely to connect with their GP, approaching the clinical encounter as a means to receive *concrete* help such as a referral or medications. Assuming that male patients seek help for different reasons than women, Colette further underlined the presumed, intrinsic gender qualities in relation to healthcare provision: ‘*I think females are often having to go to GP's for sort of like women's health things and stuff like that, like there is just that kind of bit more a consistent relationship with a GP*’. (Colette) In other words, a normative construction of masculinity and how it entangles with regular healthcare experiences is presumed to prevent men from reaching out and, in turn, prevents the concern from becoming made as recognisable for care and intervention.

Generalisations or imaginaries regarding gender and what happens outside the clinical settings were mentioned also in the context of how men come forward to seek help. Rebeka, a general practitioner with 10 years of experience, accentuated:

they just will not want to talk about it, the patients are probably less likely to ask for help because male patients generally are less likely to ask for help and even the adults they are coming and going, “oh my wife had told me to come in for a check‐up” and then you start asking and there is like five different issues that most females would have come in to sort out that they have just not been dealing with.

(Rebeka)

Rebeka's story portrayed a potential clinical encounter where males may not feel comfortable to speak about their eating patterns or dieting. She speculated that men, generally, are more hesitant to open up about these things. In Rebeka's story, there is an underlying assumption that men are more reluctant to ask for help because it is not masculine to do so. However, clinical interventions rely on an object being made and *recognised* as a particular thing, an eating disorder, in order to act on it and care for it. If the concern is driven by gender normative presuppositions, it places limits around what is and is not able to be done in clinical practice, placing barriers around care.

Barriers thought to be triggered by binary gender construction also posed difficulties in assessing the risks and diagnosing the potential disorder. John, a dietician working on eating disorders for over 5 years, explained:

Yes, the other male clients, they had quite a lot of shame about their eating disorder, and it maybe just didn’t fit with other presentations and professionals' experiences of an eating disorder, so, they found it very difficult to engage and actually seek support because it was like will people believe I have an eating disorder if it’s not as obvious as it is in females.

(John)

John's account combined shame with limited experience and unusual presentations that may challenge the imaginaries of health professionals and their clients. He emphasised how shame is associated with the perceived risk of having a ‘female’ condition and how this might constrain men from getting care. In fact, shame and stigma are effects of enacting an eating disorder in that way. Subsequently, in John's account, even when the problems become more recognisable by being brought into the clinical settings, he constructed them as difficult to address, capture and contain due to these dichotomous gendered assumptions.

Further into our conversation, I asked John if he recalled any particularly interesting male client he had. He recounted a case of a fit and athletic patient: ‘*He was relatively athletic, wasn't underweight, and played football. Those type of people we just don't hear or see about having an eating disorder*’. (John) He implied that his identity, lifestyle and gender combined produced a barrier for the realisation of risks of a potential condition. The female ‘obviousness’ of symptoms that John mentioned is crucial here to understand how presumptions about gender disrupt the classification of eating disorders for men as a medical—and therefore treatable—entity. In this case, the construction of masculinity through fitness and athleticism obscures the perception of eating disorders. Clinicians performed these characteristics as too unconventional or maybe not ‘obvious’ enough. Therefore, in their view, men that present with them may not be accurately assessed and thus overlooked.

On the one hand, clinicians' accounts of presumed ‘barriers’ to care also continue to illuminate an ‘elsewhereness’: a moment where MEDs are unrecognised as medical entities but otherwise present yet not finding a way to a clinician's office. In this way, barriers affect the ways in which MEDs are becoming ‘real’ or classified. Barriers thus disrupt and slow down the coordination of elements potentiating MEDs as a clinically *recognised* disorder (Mol 2002). As a result, in clinical practices, male eating disorders emerge in assemblages where they are not enacted as ‘real’ but rather negotiated by clinicians as fractional (Law and Singleton 2005). That is, in the discussed clinicians' accounts, the disorder emerges as not coherent, visible and classified (Jutel 2011). It appears to be something that cannot be sustained in its entirety.

### 3.3 Emergence Through Diagnostic Devices

To this point, I was interested in how MEDs are enacted as noticeable before clinicians deploy assessment and diagnostic tools and protocols. However, it is important to ask: What are the means through which clinicians may make MEDs *recognisable* and diagnostically viable? Especially considering how Jutel (2011) theorises diagnosis as a classification tool that navigates clinical care. In respect to diagnosing eating disorders, there is already a vast range of tests and indicators that assist with assessing patients—from the body mass index calculator (Maralani and McKee 2017) through blood or hormone panels to the Eating Disorder Examination Questionnaire (EDE‐Q; Mond et al. 2004). Through these, clinicians can assert and monitor with more apparent certainty what is at stake. The results of these tests do performative work to reduce doubt and uncertainty, allowing for the diagnosis—that is, helping make an object more recognisable for intervention and care. In the interviews, I asked clinicians what they use when assessing a problem or screening for a disorder among male clients.

Matthew, a dietician with 5 years of professional experience, explained:

Yeah, I think for working with men with an eating disorder you know a lot of the tools and like the assessment tools and that kind of stuff that we have got have been developed with women in mind and so sometimes it can be a little bit tricky trying to fit them to a male population you know.

(Matthew)

In his reasoning, standardised assessment tools make up MEDs but not always as stable, grounded and coherent with concordance to the preset standards. He continued:

Yes, EDE‐Q is probably a good example so for clients who engage in, because that works on you know for the last 28 days I think, last four weeks, you know, how many times have you engaged in these behaviours for some of the male clients that I have worked with, that will look very different if they are engaging in like bulking and cutting type practices then some of those things will or won’t show up depending on where they are at.

(Matthew)

Matthew posited MEDs as an elusive object that may resist being stabilised through this questionnaire. Pointing to practices such as bulking and cutting, he highlights how men do not fit into what the standard protocols are designed to monitor. Maleness, rendered here through ‘bulking and cutting’, does not coincide with the presumed ‘women in mind’ design of these diagnostic tools. Matthew's account of diagnostic practices illustrates the limits of this assessment inventory in making MEDs ‘knowable’ and therefore clinically manageable. The concern men might present with resists being pinned down through the assessment tool, making the becoming of MEDs ‘inside’ clinical settings still unfinished and fractional.

### 3.4 Emergence in Inpatient Units

So far, I have traced how MEDs are enacted before they are classified as a clinical entity and how they slipped through the traditional accessing and diagnostic tools. I have also analysed how maleness in eating disorders is enacted in participants' accounts in relation to gender and how this is said to create barriers to clinical care. Here, I go on to explore the enactments of MEDs in clinical situations once they are diagnosed as a disorder and managed in inpatient units. While doing so, I am mindful that so far, qualitative studies on male eating disorders signalled the problems of admission (Lyons et al. 2019) or broader problems that men face in order to receive care (P. J. Hay et al. 2005; K. Hay et al. 2019; Sangha et al. 2019). In other words, here I investigate how MEDs are fractional clinical objects. I also further trace how a gender dichotomy is enacted in this set of clinicians' accounts of eating disorders.

Describing the inpatient unit environment, Jackie and April highlighted how the male presence affects the default architecture of care. Jackie, a GP working with eating disordered patients for more than 20 years, said:

before we actually got him admitted as an inpatient but the issue there was that he was the only male doing the programme and it was all very skewed towards females and so he found that not very helpful at all,

(Jackie)

Jackie's patient found himself out of place, being the only male admitted at that time. His appearance challenged the default setting, which is perceived to be ‘skewed towards females’. Thus, Jackie construed the hospital arrangement as not entirely accommodating while imagining how a male patient could feel:

suppose when you think about it, if it’s a six bed or an eight bed unit then you are the token male, that it’s going to be very difficult to feel included in a group anyway and there is a lot of group work, you know, they do a lot of group work in these inpatient programs, so it might be that when they are doing their one‐on‐one it’s okay, but when they have got to get into those group situations.

(Jackie)

Being an exception in group programmes, maleness disrupts established and maintained arrangements. Jackie speculated that ‘being the token male’ might be ‘very difficult to feel included in a group’, hence she saw maleness as posing complications for effective treatment delivery regarding patients' inclusion. Russell and Laszlo suggested that men may feel like ‘lone wolves’ in treatment centres (Russell and Laszlo 2013). Here, I extend this research adding the problem of gender in delivering adequate care. For Jackie, problematised group therapies along gender lines are another moment and space where men may feel out of place. April, a registered nurse, had similar observations about that:

so and even in groups, you know there have been suggestions in the community meeting itself, for more of variety in the groups that we run, because in the past it’s been like pampering group and nail polish group and kind of activities.

(April)

April mentioned particular locations, times and activities that she thought men may not fully appreciate, framing the problem of inclusion more concretely through particular group activities. This is why she also directly mentioned ‘male inpatient units’ that could be a more inclusive place for male patients.

Furthermore, her account performs, once more, the perceived importance of gender—here in relation to the inpatient organisation of care that might better accommodate the male patient. In other words, classified, diagnosed and ‘real’ MEDs that are brought into a treatment centre are disruptive, illuminating the limits of medical interventions. Importantly, she pointed out that the limits of the current arrangements are being picked up by the staff. Hence, it is not only patients bringing attention to this problem, which brings new light to what has been previously claimed about health services not recognising the possibility of MEDs as an issue for men (Drummond 2002; MacLean et al. 2015; Räisänen and Hunt 2014).

Matthew spoke of another issue related to inclusion—a potential sense of isolation for men in the configuration of the ward:

When I was in inpatient treatment, we have often at most maybe one guy in at a time and it can be you know little bit isolating I think for people when they feel like nobody here really understands my experience or I don't see my experience reflected in the people that I am around, so there is definitely a need for more targeted therapeutic treatment options that kind of really respond to the challenges that men face.

(Matthew)

For Matthew, isolation and loneliness were seen as effects of gendered relations, primarily because men entering the inpatient environment found themselves in an almost exclusively women space. He also, as the previous accounts, presented the female‐oriented interior and spatial design as magnifying the experience of isolation. Maleness, not hanging together well with all the ‘feminine’ elements, disrupts the coordination of the network of care (Mol 2002).

I notice then the certain uniqueness of male presence in these accounts. It materialises through a fractional becoming in the inpatient units because ‘standard’ presence is assumed to be feminine (Lyons et al. 2019; McVittie et al. 2005). Therefore, clinicians perform assemblages around femaleness as the fixed arrangement. Contrastingly, male presence constitutes an incompleteness when it is emerging in these settings. As Katrina accentuated more: ‘*Alone, in the women setting. Everything prepared and imagined for a female patient, stereotypically: language, design of the ED wards, how do room look like what is in them, what do they do in a free time*’. (Katrina)

To alter the feminised treatment environments, April imagined a different way of giving space to ‘maleness’ or changing the current arrangement as a key point for the effective care delivery. As she asserted:

I’m trying to think about what else we did with those particular patients … I think some of them, I think we gave them some time on one of the other floors, so that they could … so when we didn’t have an Xbox, could go down and play because it was one of their requests and we got one. Yeah.

(April)

An Xbox becomes a token of male presence and transition to a better arranged space—an artefact through which maleness is becoming more ‘real’ in April's ward. ‘Real’ not only as a disordered body but also through its materialised version as a game console. During our interview, April also mentioned that playing a video game would be even good for men and ‘maybe not so great for some females’, making yet another distinction and defining what would be feminine and masculine regarding the design of the treatment centre.

In these accounts, gender is enacted not as a barrier to seeking help but rather as a set of relations requiring specific adjustments of care organisation. Hence, the partial presence of maleness triggers possibilities for the reorganisation of the ward. Clinicians very succinctly described how the interior design assumes gender specificity. They delineated what they think constitutes a feminine environment that does not accommodate male needs well. Thus, normative gender constructions delimit assemblages around eating disorders in relation to a presumed fixed female/male dichotomy, solidifying gender as a focal point of how care is attended to. This analysis thus illuminates that maleness in eating disorders is a matter out of place, existing awkwardly and fractionally on the verge of medical practices of clinical care and in inpatient units.

## 4 Discussion

In this paper, I have demonstrated how MEDs materialise as different things before they are diagnosed as an object of clinical intervention. In other words, I have interrogated how the concern exists ‘outside’ clinical settings in various assemblages. In clinical accounts, they are recognised as problems for patients; however, as clinicians state, patients do not necessarily admit to experiencing them. This poses problems for clinicians regarding acting and intervening. As a consequence, I have discussed this ‘outside’ presence in relation to the barriers to seeking help and the barriers preventing clinicians from acting even when the concern is diagnosed.

Moreover, I have examined how concerns are brought into the clinical setting to be made more recognisable and actionable because outside the clinical environment, they might not be taken seriously by an army veteran. Such a perspective bears the consequences of how detecting an eating disorder may involve reaching out to social and family groups as well and not only clinical sites of intervention.

That is why this paper takes the approach of making MEDs more ‘real’ in the clinical setting as a way to show the fractional becoming of the disorder, taking Barad's concept in a new direction. Fractional because clinicians enact MEDs with uncertainty and as challenging clinical knowledge. Thus, this situation implies that MEDs, even when they are brought into the clinical setting and are becoming more diagnostically valid, still find ways to defy medical definitions and still resist being pinned down.

Furthermore, I argue that gender appears to be a focal point of care delivery also inside clinical settings. However this time, the challenge comes from a male physical presence on the eating disorders ward. Traditionally and stereotypically understood femininity informed how the ward should look. In this article, I have confirmed what has been argued in previous critical qualitative social studies (Drummond 2002; Lyons et al. 2019; Thapliyal et al. 2020), which is that men feel isolated, misunderstood and alone in these settings and that a male presence is a form of disruption in these environments.

Expanding the discussion on men's experiences in treatment facilities, clinicians indicated that the current situation in treatment centres needs to be adjusted to cater for more than one gender. My analysis demonstrates that an appetite exists for practice change in these settings. Subsequently, I have shown how the disruption and disturbance caused by a male presence does not trigger or ignite an immediate change, perhaps except for small indications of an evolving assemblage (e.g., by purchasing an Xbox), but that other ways of doing care might nonetheless be possible. Although MEDs emerge through an outside concern that resists being translated into a clinical entity and, in some ways, resist coherence and coordination (Mol 2002), it nonetheless ‘holds together’ in some ways as an object of care in participants' accounts.

I claim that there is a need to transition away from more or less fixed categorical conceptualisations to attend to the fluidities and intricacies of an emergent object and gender as they are materialised in the specifics of their implementation events and situations. This invites an approach that explores intersections as material entanglements of becoming (Barad 2007). I use these terms to accentuate the incompleteness or fractionality of MEDs becoming a clinical entity that can be diagnostically classified (Jutel 2011). The fractional presence of MEDs could then be addressed by a way of thinking with care—care understood as a method used to think about realities, moments and situations that encompass men and eating disorders and clinical settings. Care that recognises ontological heterogeneity and allows speculation about how things could be. Care that is a political doing (Martin et al. 2015). Care that goes beyond biomedical categories of assessment, diagnosis and gender dichotomy reproduction and challenges them, as Rojas‐Navarro et al. (2023) argue.

Unpacking the problem of barriers to care provides examples of where this ontological heterogeneity and multiplicity could be located and why it is important to express it and not silence it. In other words, I opt for a care model that bridges the ‘outside’ and ‘inside’—or macro and micro—levels of health provision. Therefore, it is suggested that men would be more attuned to the possibility of having an eating disorder that did not appear as an unrecognised concern anymore. This could also have implications for how eating disorders are understood more generally and how eating disorders would be decentralised from focusing solely on either gender, welcoming gender nonconformity or fluidity.

Furthermore, if the normative understanding of gender is construed as a barrier both outside and inside clinical settings, it triggers the question: How can we overcome these barriers? This research suggests that, perhaps, positioning eating disorders outside fixed gender lines could be one of the answers. Especially considering that one of the biggest charities in the United Kingdom, BEAT, already promotes this way of thinking about eating disorders (https://www.beateatingdisorders.org.uk/). The fractional becoming of MEDs is also inextricably linked to a gender binary model that clinicians invoke in their accounts. The normative view of femaleness and maleness shows how deeply femaleness and ‘not‐femaleness’ are entrenched in contemporary healthcare provision in relation to MEDs. Namely, the enactment of eating disorders as a female condition restricts the ways that care can be delivered for male patients. Therefore, one conclusion to draw from this analysis might be the need to challenge the imagined fixed female/male dichotomy to offer a more sensitive and flexible model of care. Thus, one possible implication of my analysis is the need for new language, opening up the possibility in the field for a discussion of (male) eating disorders, to challenge the dichotomy and to welcome more careful, situated and locally produced modalities of care. (Male) eating disorders reconfigure how gender attaches to presumed disordering, shifting away from material‐discursive practices that continually reproduce differences between gender presentations and representations. Hence, in this paper, I align with previous intersectional feminist approaches that have begun to challenge a binary construction of eating disorder in relation to gender (LaMarre et al. 2022a, 2022b). To think in terms of (male) eating disorders would mean to slow down and shift the attention from gender to following the evidence in the making in clinical practices at a local level. Decentring the gender component in eating disorders would entail that assessment and diagnostic procedures could more adequately notice risks, not only among cis male and female bodies, but also among nonbinary and fluid ones. Moreover, it would indicate that we stop focusing on finding the distinctive characteristics of (male) eating disorders in comparison with females, as psychiatric and psychological studies tend to do. In turn, care could be more focused on following various bodies—trans, cis, LGBTQA+ and nonbinary—across different ethnicities and social classes and more succinctly noticing those that are at risk of developing an eating disorder. Although here, I have predominantly focused on clinicians’ experiences with cis, able‐bodied men, further research is needed to explore the situated experience of eating disorders in transgender, gay and ethnic minority men. Because a more open and situated understanding of the interactions between gender and eating disorders invites a more relational approach to care that is an open‐ended matter, a dynamic force rather than a stable and fixed tool (Martin et al. 2015). Care that cuts across the female/male dichotomy and attends to an emerging and lively object such as (male) eating disorders.

## Author Contributions

**Piotr Maron:** formal analysis, writing – original draft, conceptualization, supervision, validation, writing – review and editing.

## Ethics Statement

Aligning with the National Statement on Ethical Conduct in Human Research (2007; updated 2018), ethical approval for this study was obtained from the University of New South Wales Human Research Ethics Committee (HREC) in April 2020 (HREC approval number: HC200160).

## Conflicts of Interest

The author declares no conflicts of interest.

## Data Availability

The data that support the findings of this study are available upon request from the corresponding author. The data are not publicly available due to privacy or ethical restrictions.
